# Collision tumor of a papillary and follicular thyroid carcinoma: a case report

**DOI:** 10.1186/s13044-023-00167-3

**Published:** 2023-08-07

**Authors:** Kanako Kawasaki, Keita Kai, Nariyuki Tanaka, Shinichi Kido, Arisa Ibi, Akimichi Minesaki, Moriyasu Yamauchi, Yuichiro Kuratomi, Shinichi Aishima, Masahiro Nakashima, Masahiro Ito

**Affiliations:** 1 Department of Pathology & Microbiology, Saga University Faculty of Medicine, Saga, Japan; 2 Department of Otolaryngology - Head & Neck Surgery, Saga University Faculty of Medicine, Saga, Japan; 3 Department of Pathology, Saga University Hospital, Nabeshima 5-1-1, Saga, 849-8501 Japan; 4 Department of Tumor and Diagnostic Pathology, Atomic Bomb Disease Institute, Nagasaki University, Nagasaki, Japan; 5 Department of Pathology, National Hospital Organization Nagasaki Medical Center, Omura, Japan

**Keywords:** Papillary carcinoma, Follicular carcinoma, Collision tumor, BRAF, NRAS, TERT

## Abstract

**Background:**

Papillary thyroid carcinoma (PTC) and follicular thyroid carcinoma (FTC) are common differentiated thyroid cancers, but the detection of a collision tumor is an extremely rare event.

**Case presentation:**

The patient was a 69-year-old Japanese female with multiple cervical lymph node swellings and a thyroid tumor. Preoperative fine needle aspiration cytology of the enlarged lymph node revealed a cytological diagnosis of papillary thyroid carcinoma (PTC). A total thyroidectomy, right cervical dissection and paratracheal dissection were performed. Histopathological and immunohistochemical analyses of resected specimens revealed a collision tumor of PTC and FTC. Multiple metastases of papillary carcinoma were found in the dissected lymph nodes. In the PTC lesion, IHC for BRAF (V600E) was positive but negative for the FTC lesion. Genetic analyses further revealed a TERT promoter C228T mutation in PTC and a NRAS codon 61 mutation in FTC. The patient died of recurrent cancer 8 months after surgery.

**Conclusions:**

A case of a collision tumor of PTC and FTC is very rare, and even fewer cases have been subjected to genetic scrutiny. The present case was successfully diagnosed by pathological examination using immunohistochemical and genetic analyses. The TERT promoter mutation in the PTC lesion was consistent with the aggressive behavior of the cancer.

## Background

Papillary thyroid carcinoma (PTC) and follicular thyroid carcinoma (FTC) are both differentiated types of thyroid cancers derived from thyroid follicular cells. PTC is the most common accounting for 75–80% of cases, and FTC is the second most common accounting for about 10% of all thyroid cancers [1]. However, a collision tumor or synchrnous ocuurrence of PTC and FTC is extremely rare. To the best of our knowledge, only fifteen cases have been published in the English literature [2–11]. The present case was a report of a collision tumor of PTC and FTC, which exhibited different histology, phenotype and genetic alterations in the tumor tissue.

## Case presentation

**Fig. 1:**
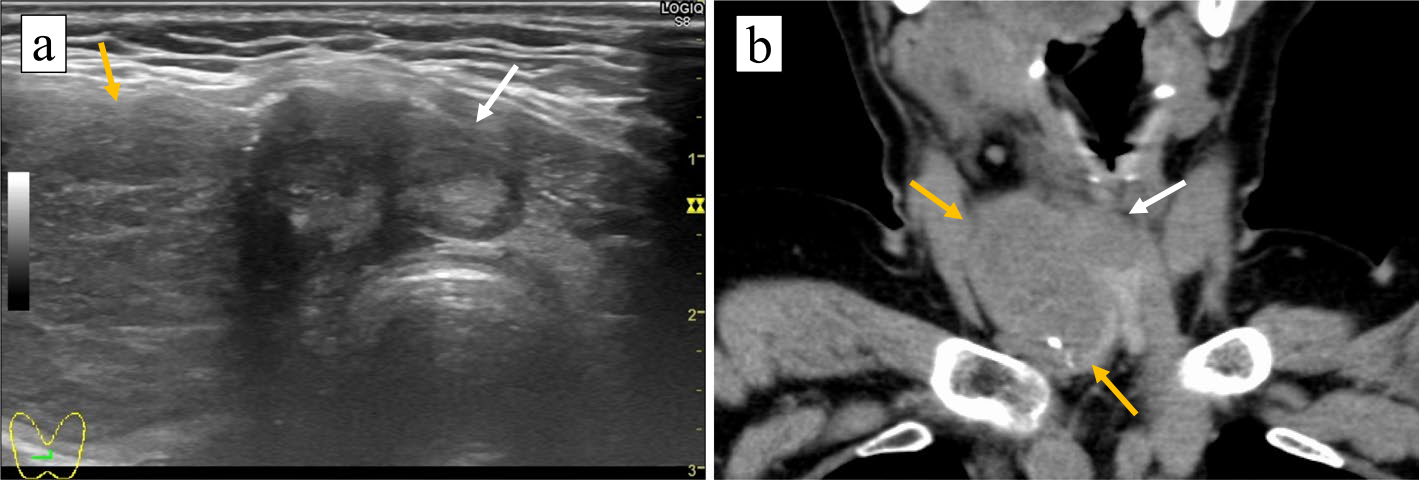
**a** Representative imager of ultrasonography. A well-defined mass lesion in the right lobe of the thyroid gland (yellow arrow) and an indistinct mass lesion in the isthmus (white arrow) are demonstrated. **b** Representative image of CT scan. The tumor of right lobe of the thyroid gland (yellow arrow) and mass lesion in the isthmus (white arrow) are demonstrated

A 69-year-old Japanese woman suffering from dysphagia visited a nearby hospital. She became aware of a right cervical mass 2 months ago and of the mass lesion becoming enlarged when dysphagia subsequently appeared. She had a history of asthma and hypertension, but no family history of thyroid disease. She never smoked and did not have an habitual alcoholic drink. Laryngoscopy revealed right vocal cord paralysis and ultrasonography revealed a well-defined mass in the right lobe of the thyroid gland and an indistinct mass in the isthmus (Fig. 1a). The CT scan showed mutiple cervical lymph node swellings and two tumors in the right lobe and isthmus of the thyroid (Fig. 1b), which were approximately 4 cm and 2 cm in diameter, respectively. The patient was transfered to our hospital for further examination and treatment. Preoperative fine needle aspiration cytology was performed on the enlarged right cervical lymph node and the cytological diagnosis was “ PTC” which is classified as Category VI in the Bethesda system [12]. No distant metastasis was found in the preoperative workup. Under the clinical diagnosis of PTC with multiple lymph node metastases, the patient underwent a total thyroidectomy, right neck dissection and bilateral paratracheal dissection. No residual tumor was intraoperatively recognized. The resected specimens were submitted for histopathological examination.

**Fig. 2:**
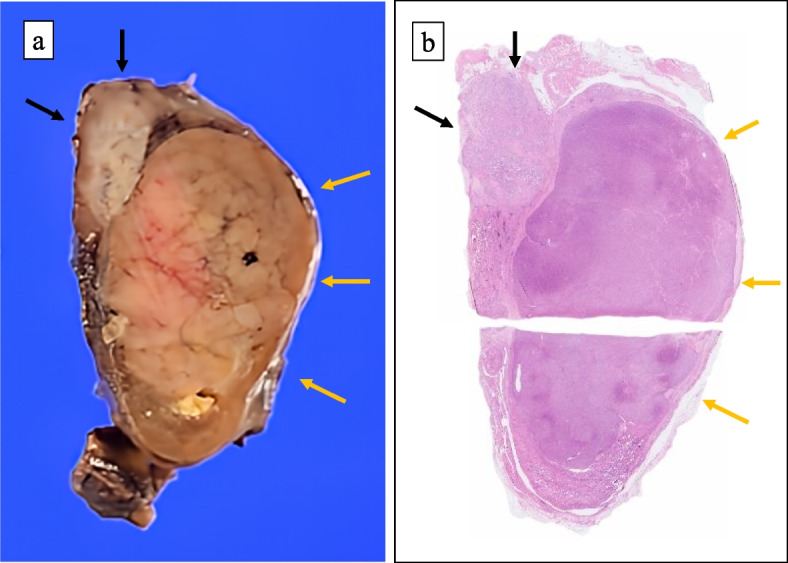
**a** Gross appearance of the cut surface of the tumor. **b** Loupe image of glass slide of hematoxylin and eosin staining. The lesion of PTC is indicated as black arrow and the lesion FTC is indicated as yellow arrow

**Fig. 3:**
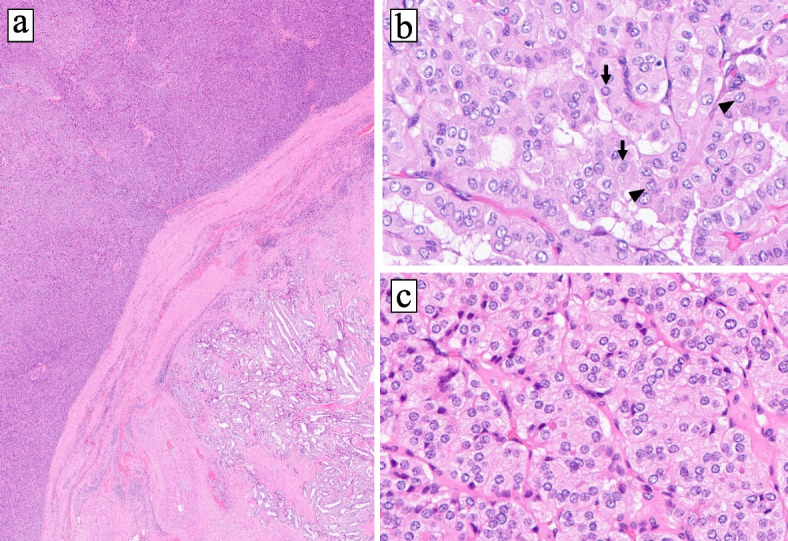
Microscopic images, **a** The histological image of low-magnification. The left-upper side of image is FTC lesion and right-lower side of image is PTC lesion (× 20, hematoxylin–eosin). **b** High-magnification image of papillary carcinoma lesion. Typical nuclear findings of PTC, such as ground-glass nuclei, nuclear grooves (arrow heads), and pseudo-inclisions (arrows) are observed (× 400, hematoxylin–eosin). **c** High-magnification image of FTC lesion. Typical nuclear findings of PTC are not observed (× 400, hematoxylin–eosin)

**Fig. 4:**
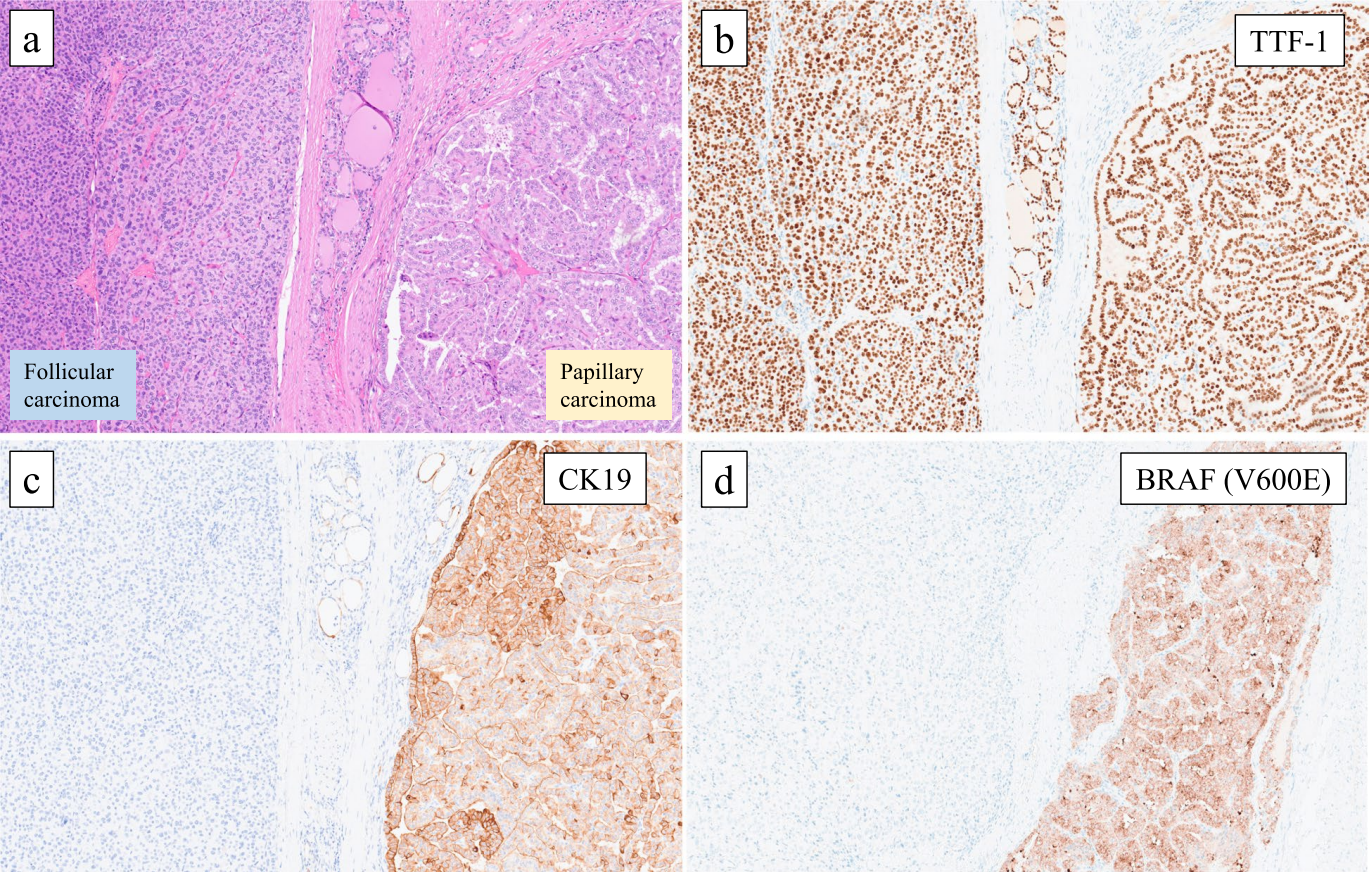
Findings of IHC. **a** The image of hematoxylin–eosin staining. The left side is FTC, and the right side is PTC (× 100). **b** IHC of TTF-1 (× 100). Both of FTC and PTC are positively stained. **c** IHC of CK19 (× 100). The tumor cells of PTC are positively stained whereas the tumor cells of FTC are negative. **d** IHC of BRAF (V600E) (× 100). The tumor cells of PTC are positively stained whereas the tumor cells of FTC are negative

**Fig. 5:**
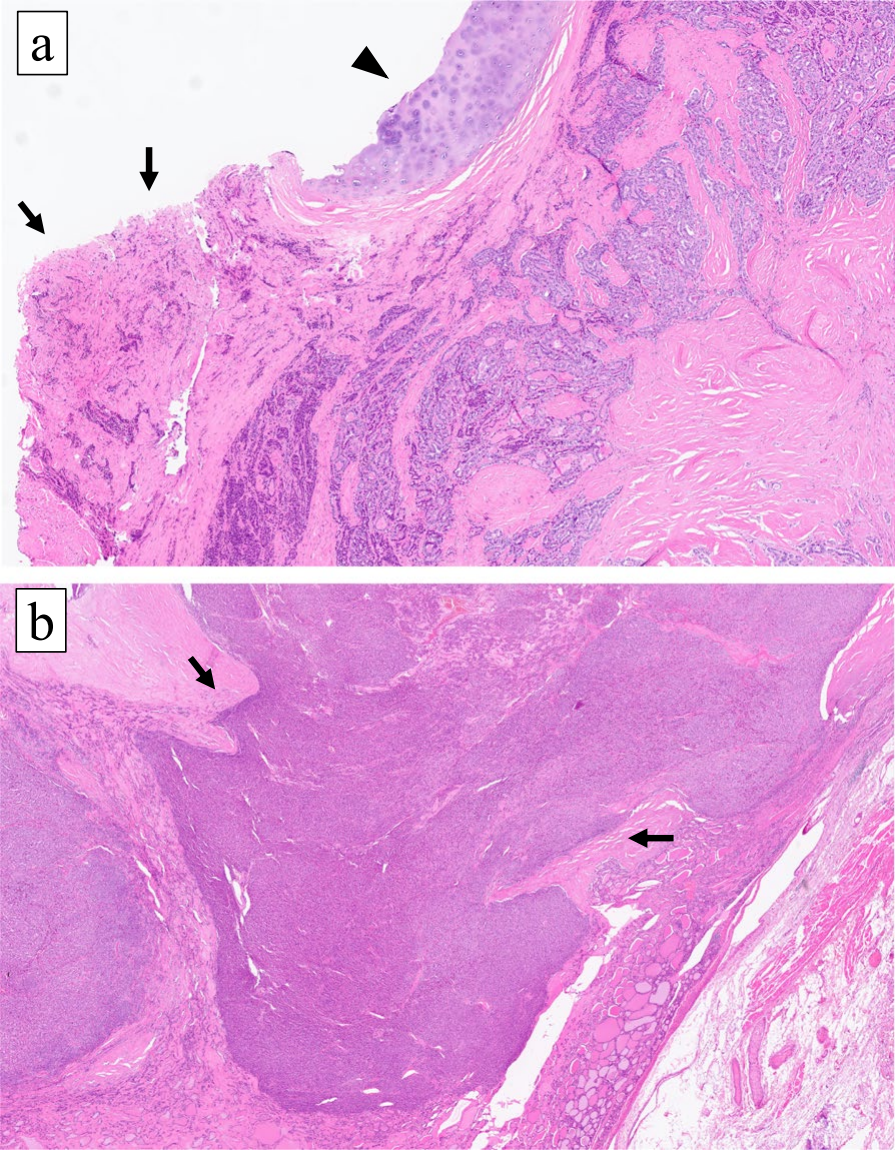
**a** The image of invasion of PTC to the inferior pharyngeal constrictor muscle (× 20, hematoxylin–eosin). Arrowhead: partially resected cricoid cartilage. Arrows: surgical ablation margin which was judged to be positive. **b** The image of capsular invasion of FTC (× 20, hematoxylin–eosin). Arrows: the tumor capsule

The cut surfase of the tumor grossly exhibited yellowish-brown and whitish masses (Fig. 2a, b**)**. Histologically, the whitish mass showed papillary or follicular growth of tumor cells with ground-glass nuclei and some nuclear grooves and pseudo-inclusions (Fig. 3a, b). Immunohistochemistry (IHC) revealed that the tumor cells in this lesion were positive for TTF-1, thyroglobulin, CK19, HBME-1, Galectin-3 and BRAF (V600E). The Ki-67 labeling index was 5% (Fig. 4). The tumor had extended beyond the thyroid capsule and invaded the inferior pharyngeal constrictor muscle and thus was considered as pT4a in the TNM classification (Fig. 5a). The surgical margin was positive at the invasion site of the inferior pharyngeal constrictor muscle. No necrosis was observed and the mitotic index of this lesion was 2 / 10HPF, thus the tumor of this lesion did not fit the criteria of differentiated high-grade thyroid carcinomas (DHGTC) [13]. The tumor of this lesion was pathologically diagnosed as an infiltrative follicular variant of PTC in the 2022 WHO classification of thyroid tumors [13]. Dissected lymph nodes showed multiple metastases (20 / 32) of the PTC includng unilateral Level V lymph nodes (pN1b). Genetic analyses for NRAS codon 61 mutation and TERT promoter mutation were performed by employing the droplet digital PCR method using the DNA extracted from formalin-fixed and paraffin-embedded tumor tissues, as previously described by one of the co-authors [14]. The results of genetic analyses further revealed a TERT promoter C228T mutation, but no NRAS mutation was detected.

In contrast, histologically the yellowish-brown mass showed that the tumor cells had an eosinophilic cytoplasm and round to oval shaped nuclei with granular chromatin. The tumor proliferated trabecular or solid, and formed a peritumoral fibrous capsule. Typical nuclear findings suggesting a PTC were not observed. The tumor exhibited predominantly expanding growth and capsular invasion was detected (Fig. 5b). No necrosis was observed and the mitotic index of this lesion was less than 1 / 10HPF, thus the tumor of this lesion did not fit the criteria of DHGTC [13]. In IHC, the tumor cells were positive for TTF-1 and thyroglobulin, but negative for CK19, HBME-1, Galectin-1 and BRAF (V600E). The Ki-67 labeling index was 3% (Fig. 4). Based on nuclear findings, the presence of capsular invasion and IHC results, the tumor comprising this lesion was pathologically diagnosed as minimally invasive FTC (mi-FTC) in the 2022 WHO classification [13]. Genetic analysis for this FTC lesion futher revealed a NRAS codon 61 mutation, but a TERT promoter mutation was not detected. The results of IHC and genetic analyses for both PTC and FTC lesions are summerized in Table 1.

**Table 1:** Results of IHC and genetic analysis in both tumors

	PTC	FTC
CK AE1/AE3, IHC	+	+
TTF-1, IHC	+	+
Thyroglobulin, IHC	+	+
CK19, IHC	+	-
HBME-1, IHC	+	-
Galectin-3, IHC	+	-
BRAFV600E, IHC	+	-
Ki-67 LI, IHC	5%	3%
TERT promoter mutation	+ (C228T)	-
NRAS mutation	-	+ (codon 61)

*PTC* Papillary thyroid carcinoma, *FTC* Follicular thyroid carcinoma, *IHC* immunohistochemistry

Based on the above results, this case was finally diagnosed as a collision tumor of PTC and FTC, each stage at surgery being pT4aN1b and pT3aN0, respectively. The patient was scheduled to receive radioactive iodine therapy. However, she declined to receive the adjuvant therapy. Eight months after surgery, recurrent tumor was clinically detected as a rapidly growing cervical mass. The patient did not accept further clinical examination nor treatment and died of recurrent tumor 8 months after surgery. Suffocation due to airway stenosis by tumor growth was considered as the direct cause of death.

## Disccusion and conclusions

The collision tumor is defined as a neoplastic lesion comprised of two or more distinct cell populations that maintain distinct borders [15]. Therefore, the present case is distinctly categorized as collision tumor. The co-existence of PTC and FTC is extremely rare. The first case of a co-existence of PTC and FTC was reported by Plauche et al. in 2013 as collision tumor of PTC and FTC [2]. Since then, 16 cases of co-existence of PTC and FTC including our case have been reported in the English literature [2–11]. The reported series of co-existence of PTC and FTC are summerised in Table 2. The average age of the patients was 53.4 years (range: 12 to 79) and female predominance (11 of 16 cases) was noted. Of these, metastases were found in 6 cases and no tumor-associated fatal case have been reported except for the present case. Genetic alterations have been analyzed in 4 cases. Of these, BRAF mutations in the PTC lesion and NRAS mutations in the FTC lesion have been reported [3, 11]. TERT promoter mutation was examined in only 1 case and no mutation was detected [11]. The present case clearly demonstrated differences in the PTC and FTC lesions in terms of the morphology, immunophenotype and genetic alterations, including a TERT promoter mutation.

**Table 2:** Summary of the reported case series of co-existence of PTC and FTC

Case No	Author	Age (years)	Sex	Size, location of PTC	Size, location of FTC	Lymph node metastasis	Distant metastasis	Immunohistochemistry	Genetic analysis	Outcome
1	Plauche V, et al. [2]	62	F	multifocal, largest 1 cm, left lobe	4.1 cm, left lobe and 0.3 cm right lobe	none	none	N/A	N/A	N/A
2	Cracolici V, et al. [3]	63	F	1.7 cm, left lobe	1.1 cm, right lobe	metastasis of PTC	metastasis of FTC to the 11th rib	positive for TTF-1 and thyroglobulin, negative for calcitonin and BRAF	BRAF(V600E) mutation in PTC, NRAS(Q61R) mutation in FTC	liver and T12 metastasis, alive for at least 6 months
3	Dai DJ, et al. [4]	66	F	0.5 cm, left lobe	1.2 cm, right lobe	none	none	N/A	N/A	N/A
4	He X, et al. [5]	71	M	0.1 cm, left lobe	4.8 cm, right lobe	none	metastasis of FTC to right adrenal grand	positive for thyroglobulin	N/A	alive for at least 14 months after thyroidectomy
5	Pishdad R, et al. [6]	79	M	left / right and size unknown	left / right and size unknown	none	metastasis of FTC to left femur	N/A	no RAS mutation	alive for at least 7 months after treatments
6	Abdelaal A, et al. [7]	31	F	5 cm, left lobe	1.3 cm, left lobe	none	none	N/A	N/A	alive for at least 2 years
7	Abdelaal A, et al. [7]	61	M	0.3 mm, right lobe	6 cm, right lobe	none	none	N/A	N/A	alive for at least 15 months
8	Abdelaal A, et al. [7]	59	M	1.5 cm, left lobe or isthmus	5 cm, left lobe and isthmus	none	none	N/A	N/A	alive for at least 22 months
9	Abdelaal A, et al. [7]	56	F	multifocal, both lobes, largest 1 cm	4.5 cm, left lobe	none	none	N/A	N/A	lost to follow-up after surgery
10	Abdelaal A, et al. [7]	35	F	two foci, largest 0.8 cm, right lobe	1.3 cm, right lobe	none	none	N/A	N/A	lost to follow-up after treatment
11	Abdelaal A, et al. [7]	52	F	0.8 cm, right lobe	2.7 cm, right lobe	none	none	N/A	N/A	alive, unknown for follow-up period
12	Feng JW, et al. [8]	40	F	1.8 cm, right lobe	left lobe, unknown for size	none	none	In FTC, positive for CK19, thyroglobulin, negative for calcitonin, Galectin-3, Chromogranin A, Synaptophysin, CD56 and TIF-1, Ki-67LI was 1%	N/A	alive at least 26 months
13	Vlaenderen JV, et al. [9]	12	F	several mm, left lobe	2.9 cm, left lobe	none	none	in PTC, positive for HBME	No mutation is found in BRAF, NRAS, RET/PTC rearrangements, TSHR gene, GNAS gene and PTEN gene	N/A
14	Carrion AMS, et al. [10]	56	M	2.5 cm, right lobe	1.5 cm, left lobe	LN metastasis of PTC	right shoulder bone metastasis	N/A	N/A	N/A
15	Stenman A, et al. [11]	43	F	3 cm, right lobe	1.2 cm, right lobe	central and lateral LN metastasis of PTC, lateral LN metastasis of FTC	none	In PTC, positive for BRAF1, 50% of tumor cells positive for thyroglobulin and Ki-67 LI was 5.6%. in FTC, negative for BRAF1 and Ki-67 LI was 3%	p.Q61R missense NRAS mutation was detected in both primary and LN metastasis of FTC. TERT promoter C228 and C250 were both wild type in FTC	alive, unknown for follow-up period
16	Present case	69	F	5.2 cm, right lobe	4.0 cm, right lobe and isthmus	central and lateral lymph node metastasis of PTC	none	In PTC, positive for TTF-1, thyroglobulin, CK19, HBME-1, Galectin-3 and BRAF (V600E), and the Ki-67LI was 5%. In FTC, positive for TTF-1 and thyroglobulin, and negative for CK19, HBME-1, Galectin-1 and BRAF (V600E), Ki-67 LI was 3%	In PTC, TERT promoter C228T mutation was detected whereas NRAS mutation was not detected. In FTC, TERT promoter mutation was not detected whereas NRAS codon 61 mutation was detected	Died of recurrent tumor 8 months after surgery

*PTC* Papillary thyroid carcinoma, *FTC* Follicular thyroid carcinoma, *Ki-67LI* Ki-67 labeling index

The BRAF (V600E) mutation is the most frequently detected genetic mutation in PTC and is found in 35–80% of adult cases of PTC [16–19]. The substitution of the 600th codon from valine to glutamate causes sustained BRAF activation. BRAF mutation-positive PTC have been reported to have a higher frequency of extra-thyroidal extension, lymph node metastasis and tumor recurrence, which has been correlated with tumor death [19]. RAS mutations are associated with the development of many cancers and are detected in 40–60% of FTC and 20–40% of follicular adenomas in the thyroid gland. The most frequent is the NRASQ61R mutation; BRAF and RAS mutations are mutually exclusive. HRAS and KRAS mutations in FTC have also been reported [20, 21]. Taken together, PTC is tipically BRAF-driven and FTC is tipically RAS-driven [13]. As previously documented, the collesion / co-existence of PTC and FTC is extremely rare. It is ideal that definite pathological diagnosis has done not only morphological findings but also genetic finding of BRAF and RAS (tipically NRAS codon 61) mutation when the collesion / co-existence of PTC and FTC is histlogically suspected.

TERT promoter mutations are genomic abnormalities in thyroid cancer, and are reported to occur in 5–15% of PTC. Papillary carcinomas with TERT promoter mutations are usually characterized as being mainly found in older patients, having larger tumor sizes, frequent lymph node and distant metastases, advanced TNM stages, and more recurrences. TERT promoter mutations are strongly associated with their clinicopathologically aggressive features [22]. In the present case, genetic analysis revealed a TERT promoter mutation in the PTC lesion. Despite the its morphology was not met the criteria of DHGTC, the PTC lesion in the present case showed aggressive behavior, such as extra-thyroidal invasion, multiple lymph node metastases, tumor recurrence and a fatal clinical outcome. These findings were consistent with the characteristics of PTC with a TERT promoter mutation.

In conclusion, we have reported a very rare case of a collision tumor of PTC and FTC, which was successfully diagnosed by pathological examinations using IHC and genetic analyses. This is the first case of the collision / coexistence of PTC and FTC in which TERT promoter mutation is confirmed. The TERT promoter mutation in the PTC lesion accounted for its aggressive behavior.

## Data Availability

Not applicable.

## Abbreviations

PTC: Papillary thyroid carcinoma
FTC: Follicular thyroid carcinoma
IHC: Immunohistochemistry
TTF-1: Thyroid transcription factor 1
CK: Cytokeratin
HBME-1: Hector Battifora mesothelial-1
